# Editorial: Non-coding RNAs in diseases of the nervous system

**DOI:** 10.3389/fncel.2023.1226950

**Published:** 2023-06-09

**Authors:** Cristina R. Reschke, Ezharul Hoque Chowdhury, Alan Yiu Wah Lee

**Affiliations:** ^1^School of Pharmacy and Biomolecular Sciences, RCSI University of Medicine and Health Sciences, Dublin, Ireland; ^2^Jeffrey Cheah School of Medicine and Health Sciences, Monash University Malaysia, Selangor, Malaysia; ^3^School of Pharmacy and Pharmaceutical Sciences, University of Sunderland, Sunderland, United Kingdom

**Keywords:** non-coding RNAs (ncRNAs), neurodegeneration, CNS injuries, Parkinson's disease, neurological diseases, epilepsy

The bewildering observation of structural and functional proteins in the body being encoded by just a small fraction of the human genome has led to the discovery of the intriguing fact that >80% of the genome is transcribed into a diverse group of non-coding RNAs (ncRNAs) (ENCODE Project Consortium, 2012). These ncRNAs can be broadly categorized into small ncRNAs (eg. microRNAs), long ncRNAs (lncRNAs) and circular RNAs (circRNAs), which are largely involved in controlling gene expression at transcriptional, post-transcriptional or epigenetic levels (Batista and Chang, 2013). Development of the nervous system is a complex and tightly regulated process, which involves dynamic and precise control of gene expression in a spatio- and temporal-specific manner. It is therefore not surprising that >40% of ncRNAs are found to be specifically expressed in the nervous system (Derrien et al., 2012). Accumulating evidence suggests that they play critical role not only during development but also in the adult stage. Dysregulated function of ncRNA is expected to result in diseases. Emerging evidence suggests that mutation of lncRNA or a dysregulation of their expressions correlates with a variety of disorders in the nervous system, including autism spectrum disorder, amyotrophic lateral sclerosis, Alzheimer's disease, bipolar disorder, Huntington's disease, Parkinson's disease (Li et al., 2019).

One of the hallmark pathological features of Parkinson's disease (PD) is the progressive loss of dopaminergic neurons in the substantia nigra. The decline of dopamine levels in the striatum results in motor dysfunction. While the underlying causes of PD have not been completely elucidated, events such as aberrant protein aggregation and degradation, mitochondrial dysfunction, cell death by apoptosis, autophagy and ferroptosis, oxidative stress, inflammatory responses are thought to contribute to the loss of dopaminergic neurons. Interestingly, accumulating evidence suggests that lncRNAs play important roles in some of these processes, and are plausibly implicated in the pathogenesis of PD. Selvakumar et al. provided a comprehensive review of the involvement of various microRNAs (miRNAs) in the regulation of apoptosis, autophagy, mitophagy, and neuroinflammation in PD. Genes that are targeted by these miRNAs include brain-derived neurotrophic factor, leucine-rich repeat kinase 2, death-associated protein kinase 1, glutamate transporter 1, and tumor growth factor-β1. Importantly, these miRNAs also represent potential therapeutic targets and biomarkers for PD. This is exemplified by findings in an original research article by Bhattacharyya et al., who reported that the level of miR-128 was significantly decreased in exosomes derived from PD patients. In a cellular model of PD induced by 6-OHDA, a downregulation of miR-128 level was also observed. Forced expression of miR-128 was shown to provide neuroprotective effect by reducing the production of mitochondrial superoxide and inhibiting the activation of apoptosis-triggering transcription factor FoxO3a. Mechanistically, miR-128 inhibits both the intrinsic and extrinsic pathways of apoptosis by suppressing the activation of pro-apoptotic targets of FoxO3a, like FasL and PUMA. Furthermore, miR-128 prevents neurite shortening and maintains expression of the presynaptic protein Synaptophysin and the post-synaptic protein PSD-95. Taken together, miR-128 represents a promising therapeutic target and diagnostic marker for PD.

CNS injuries, such as traumatic brain injury (TBI), subarachnoid hemorrhage (SAH), intracerebral hemorrhage (ICH), cerebral ischemic stroke, and spinal cord injury (SCI) are among the leading causes of disability and mortality. Apart from primary insult, the ensuing secondary damages in CNS injuries often cause more extensive and chronic impact. Interplay between complex pathological processes such as oxidative stress, neuroinflammation, apoptosis, and autophagy results in further neuronal cell death (Ng and Lee, 2019). Recent studies showed that injury of the nervous system is associated with alterations in the expression profile of ncRNAs and is dependent on the degree of severity (Zhang and Wang, 2019). In CNS injuries, ncRNAs are implicated in an array of physiological processes, like angiogenesis, apoptosis, and inflammation via different regulatory pathways, hence playing an important role in injury-induced secondary brain damage. Pedrosa et al. reported a general overexpression of miRNAs in the cerebrospinal fluid (CSF) of patients with acute SAH, which can develop into delayed cerebral ischemia (DCI). Specifically, exceptionally high levels of hsa-miR-320e and hsa-miR-451a were observed in patients who developed post-SAH DCI. Some of the upregulated miRNAs were implicated in the regulation of pathways that control neurological damage in stroke, angiogenesis, and integrity of blood-brain barrier (BBB). In short, these findings suggest the potential use of these miRNAs as prognostic and diagnostic biomarkers in SAH patients.

It is not an uncommon observation that patients, particularly older adults suffer from varying degree of cognition dysfunction after surgery. Collectively, post-operative cognitive dysfunction (POCD) refers to varying degree of decline in learning and memory, executive function, attention, and orientation after anesthesia and surgery. Surgical trauma and anesthesia are believed to cause BBB damage, neuroinflammation, mitochondrial dysfunction, oxidative stress, neuronal apoptosis, autophagy, impaired synaptic function, and loss of neurotrophic support (Lin et al., 2020). Yang et al. presented an overview of various ncRNAs that are involved in these cellular processes. Notably, some ncRNAs have been implicated in anesthesia-induced accumulation of Aβ and hyperphosphorylation of tau, contributing to neuronal cell death and cognitive dysfunction.

Epilepsy is a common neurological condition due to abnormally excessive electrical activity in the brain, which causes frequent seizures. Prolonged and uncontrolled seizure may cause brain damages. Accumulating evidence suggests functional significance of regulatory immune cells in the modulation of epileptogenic processes and seizures (Yue et al., 2022). Sharifi et al. reported differential expression of lncRNAs that are associated with regulatory T cells (Tregs) in different forms of epilepsy. These findings not only support the involvement of Tregs in epilepsy but also the potential role of these lncRNAs as diagnostic markers for different epileptic conditions.

While ncRNAs are generally deemed to serve regulatory role in gene expression, increasing evidence suggests that some of them in fact code for small peptides (Wang et al., 2019). Since lncRNAs are specifically enriched in the brain, they are more likely to contribute to the exceptionally high population of neuropeptides in the central nervous system. Mohaupt et al. provided a timely review of the current development in alternative proteome in the nervous system, highlighting the underrated importance of alternative neuroproteome encoded by ncRNAs in various neurodegenerative diseases.

In summary, this Research Topic covered different pathological roles of ncRNAs in the nervous system. This collection of articles is expected to provide insight and contributes to the rapid development of ncRNAs as targets for therapeutic intervention and biomarkers in diseases of the nervous system.

## Author contributions

AL prepared the first draft of the editorial. EC and CR commented and revised the manuscript. All authors approved the final version for publication.

## References

[B1] Batista P. J. Chang H. Y. (2013). Long noncoding RNAs: cellular address codes in development and disease. Cell 152, 1298–1307. 10.1016/j.cell.2013.02.012 23498938 PMC3651923

[B2] Derrien T. Johnson R. Bussotti G. Tanzer A. Djebali S. Tilgner H.. (2012). The GENCODE v7 catalog of human long noncoding RNAs: analysis of their gene structure, evolution, and expression. Genome Res. 22, 1775–1789. 10.1101/gr.132159.111 22955988 PMC3431493

[B3] ENCODE Project Consortium (2012). An integrated encyclopedia of DNA elements in the human genome. Nature 489, 57–74. 10.1038/nature11247 22955616 PMC3439153

[B4] Li L. Zhuang Y. Zhao X. Li X. (2019). Long non-coding RNA in neuronal development and neurological disorders. Front. Genet. 23, 744. 10.3389/fgene.2018.00744 30728830 PMC6351443

[B5] Lin X. Chen Y. Zhang P. Chen G. Zhou Y. Yu X. (2020). The potential mechanism of postoperative cognitive dysfunction in older people. Exp. Gerontol. 130, 110791. 10.1016/j.exger.2019.110791 31765741

[B6] Ng S. Y. Lee A. Y. (2019). Traumatic brain injuries: pathophysiology and potential therapeutic targets. Front. Cell Neurosci. 27, 528. 10.3389/fncel.2019.00528 31827423 PMC6890857

[B7] Wang S. Mao C. Liu S. (2019). Peptides encoded by noncoding genes: challenges and perspectives. Signal. Transduct. Target. Ther. 13, 57. 10.1038/s41392-019-0092-3 31871775 PMC6908703

[B8] Yue J. Xu R. Yin C. Yang H. Zhang C. Zhao D. (2022). Negative effects of brain regulatory T cells depletion on epilepsy. Prog. Neurobiol. 217, 102335. 10.1016/j.pneurobio.2022.102335 35931355

[B9] Zhang L. Wang H. (2019). Long non-coding RNA in CNS injuries: a new target for therapeutic intervention. Mol. Ther. Nucleic Acids 17, 754–766. 10.1016/j.omtn.2019.07.013 31437654 PMC6709344

